# Oxygen-supplying ROS-responsive prodrug for synergistic chemotherapy and photodynamic therapy of colon cancer

**DOI:** 10.3389/fphar.2024.1325544

**Published:** 2024-02-14

**Authors:** Ying Hao, Tailuo Liu, Hao Zhou, Runhao Xu, Ka Li, Mao Chen, Yuwen Chen

**Affiliations:** ^1^ Laboratory of Heart Valve Disease, West China Hospital, Sichuan University, Chengdu, China; ^2^ West China Hospital, West China School of Nursing, Sichuan University, Chengdu, China; ^3^ Department of Cardiology, West China Hospital, Sichuan University, Chengdu, China

**Keywords:** camptothecin, pheophorbide a, platinum nanozyme, reactive oxygen species-responsive, synergistic therapy

## Abstract

**Introduction:** The synergistic treatment of chemotherapy and photodynamic therapy (PDT) has remarkable potential in cancer therapy. However, challenges remain, such as unstable chemotherapeutic drug release, suboptimal targeting, and reduced efficacy of PDT under hypoxic conditions commonly found in solid tumors.

**Methods:** To address these issues, we use camptothecin (CPT) and pheophorbide a (Pa) incorporated through the functional thioketal, which serves as the reactive oxygen species (ROS)-responsive trigger, to construct a ROS-responsive prodrug (CPT-TK-Pa). Subsequently, we co-loaded it with a platinum nanozyme (PtNP) in distearylphosphatidylethanolamine–polyethylene glycol (DSPE–PEG) to obtain the ROS-responsive prodrug nanoparticle (CPT-TK-Pa/Pt NP).

**Results and Discussion:** Specifically, the incorporated PtNP within CPT-TK-Pa/Pt NP positively catalyzes the conversion of hydrogen peroxide (H_2_O_2_) to oxygen, thereby ameliorating the hypoxic state of the tumor. This enhanced oxygen generation could replenish the oxygen that is consumed by Pa during 660 nm exposure, enabling controlled CPT release and amplifying the photodynamic response. *In vitro* investigations reveal the potency of CPT-TK-Pa/Pt NPs in inhibiting colon tumor cells. Given its ROS-responsive release mechanism and enhanced PDT efficacy, CPT-TK-Pa/Pt NP has the potential to be a promising candidate for cancer therapy.

## 1 Introduction

Colorectal cancer accounts for 10% of all cancer cases worldwide and is the third leading cause of cancer-related morbidity and mortality. While chemotherapy remains a primary therapeutic modality for colon tumors (Morton et al., 2023), the use of chemotherapeutics, such as analogs of camptothecin (CPT) like irinotecan and topotecan, faces challenges, such as low bioavailability, inadequate targeting, consequent systemic toxicity, drug resistance, and immunosuppression (Ying et al., 2023). Recent research has underscored the importance of combination therapies in improving cancer treatment outcomes (Hao et al., 2023). In particular, photodynamic therapy (PDT), a minimally invasive therapeutic approach, utilizes light to generate reactive oxygen species (ROS), causing irreversible damage to neoplasms. However, the intrinsic hypoxic nature of solid tumors impedes the PDT’s efficacy (Wan et al., 2021). Thus, there is an urgent clinical need to develop a synergistic chemophotodynamic treatment to ensure the selective release of chemotherapeutics and augment ROS production for optimal PDT application.

Prodrugs are capable of selectively releasing therapeutic agents at precise sites while minimizing systemic toxicity, which is facilitated by specific characteristics of the local microenvironment in a tumor, including variations in pH, glutathione levels, and some enzymes with higher concentrations, or external stimuli such as light, heat, and ultrasound (Yin et al., 2019). Notably, red or near-infrared light is a promising trigger for controlled drug release (Zhao et al., 2019; Lu et al., 2022) because of its high tissue penetration, low phototoxic effects, and the ability to selectively break chemical bonds. Concurrently, when exposed to 660 nm irradiation, photosensitizers can generate ROS and selectively break ROS-sensitive covalent bonds (Jia et al., 2021), including thioketal (Zhai et al., 2022), thioether (Criado-Gonzalez and Mecerreyes, 2022), diselenide ether (Hailemeskel et al., 2019), and peroxyacetate (Yao et al., 2019). Pheophorbide a (Pa), one of the products of chlorophyll degradation, has recently attracted widespread attention as a natural photosensitizer, exhibiting significant photodynamic and antitumor activities by disrupting tumor DNA integrity at high concentrations, providing a noninvasive and highly selective approach to cancer treatment (Guo et al., 2022). CPT, the chemotherapeutic agent, acts as an inhibitor of topoisomerase I, stabilizing the topoisomerase I–DNA complex, obstructing DNA duplication and transcription, and then inducing cancer cell apoptosis (Schultz, 1973). However, hypoxia remains a major barrier to the therapeutic efficacy of current photodynamic therapy. To address these challenges, strategies involving *in situ* oxygen generation and exogenous oxygen delivery have been advanced (Sharma et al., 2019; Xu et al., 2020). However, concerns persist, such as the instability and rapid consumption of oxygen-generating substances, unintended oxygen leakage, and suboptimal exogenous oxygen yield, which limit the therapeutic potential of PDT (Wan et al., 2021). We have prepared a platinum nanozyme (PtNP) with remarkable stability and catalytic activity in decomposing H_2_O_2_ to oxygen, which ameliorated the hypoxic condition characteristic of tumor tissues and sustainably replenished the oxygen consumed by PDT, indicating its relevance in PDT applications (Hao et al., 2020; Hao et al., 2023).

**FIGURE 1:**
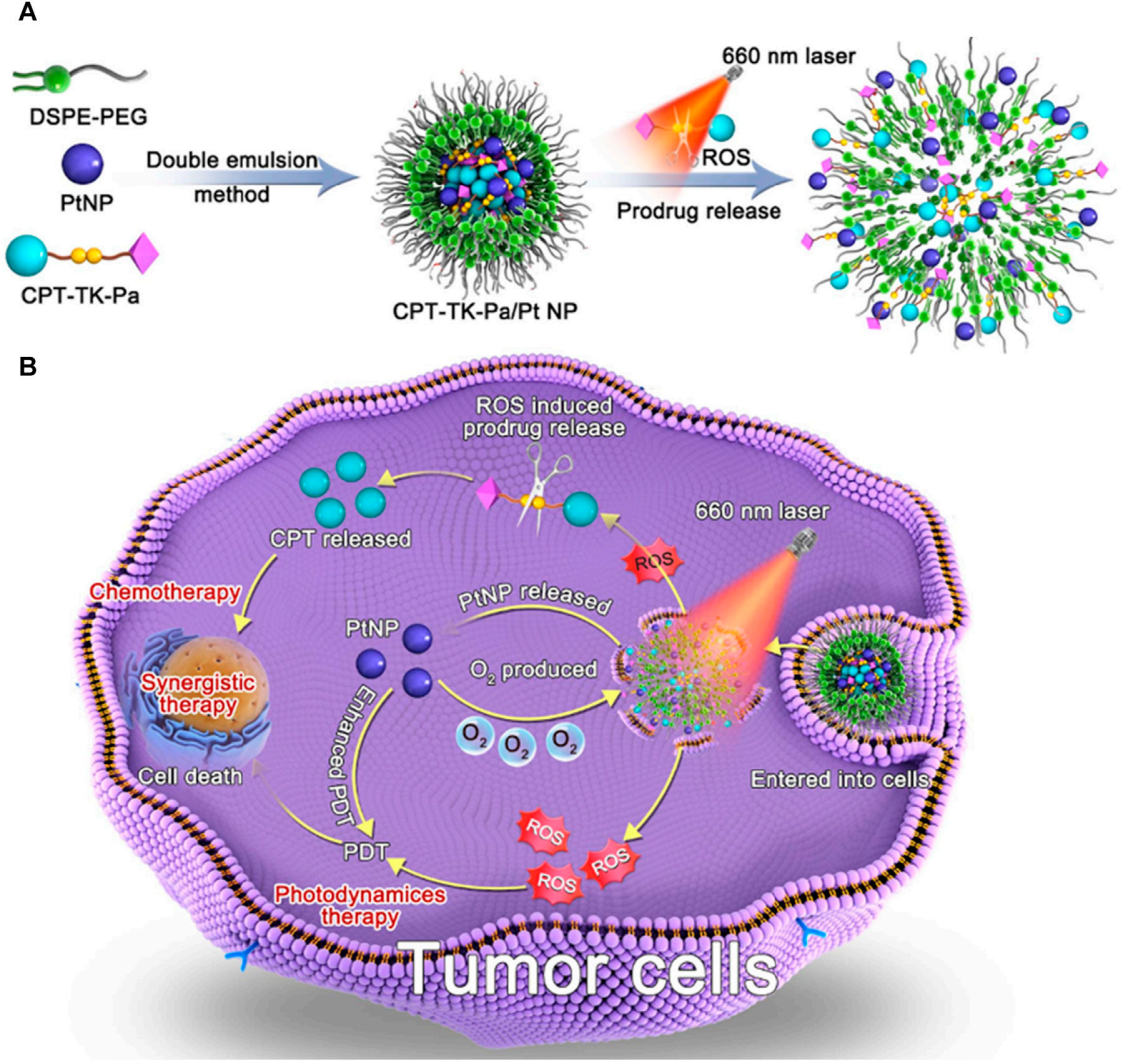
Schematic illustration of **(A)** preparation of CPT-TK-Pa/Pt NP and **(B)** colon cancer treatment.

In this study, we achieved a ROS-responsive prodrug (CPT-TK-Pa) by linking CPT and Pa via a thioketal bond and then used distearylphosphatidylethanolamine-polyethylene glycol (DSPE–PEG) to load CPT-TK-Pa and PtNP to obtain the ROS-responsive nanoprodrug (CPT-TK-Pa/Pt NP), which improved the chemophotodynamics in colon cancer therapy. As presented in Figure 1, the prodrug generated ROS under 660 nm irradiation, which enhanced the efficacy of PDT and controlled the release behavior of embedded CPT. This oxygen generation counteracted the oxygen consumption induced by Pa under 660 nm irradiation, ensuring the precise on-demand liberation of CPT and strengthening the effect of PDT. Based on its ideal ROS-responsive drug release mechanism and superior PDT efficiency, the innovatively designed CPT-TK-Pa/Pt NP may hold promise as a satisfactory approach in clinical intervention for colon cancer.

## 2 Results and discussion

### 2.1 Synthesis and characterization of CPT-TK-Pa

**FIGURE 2:**
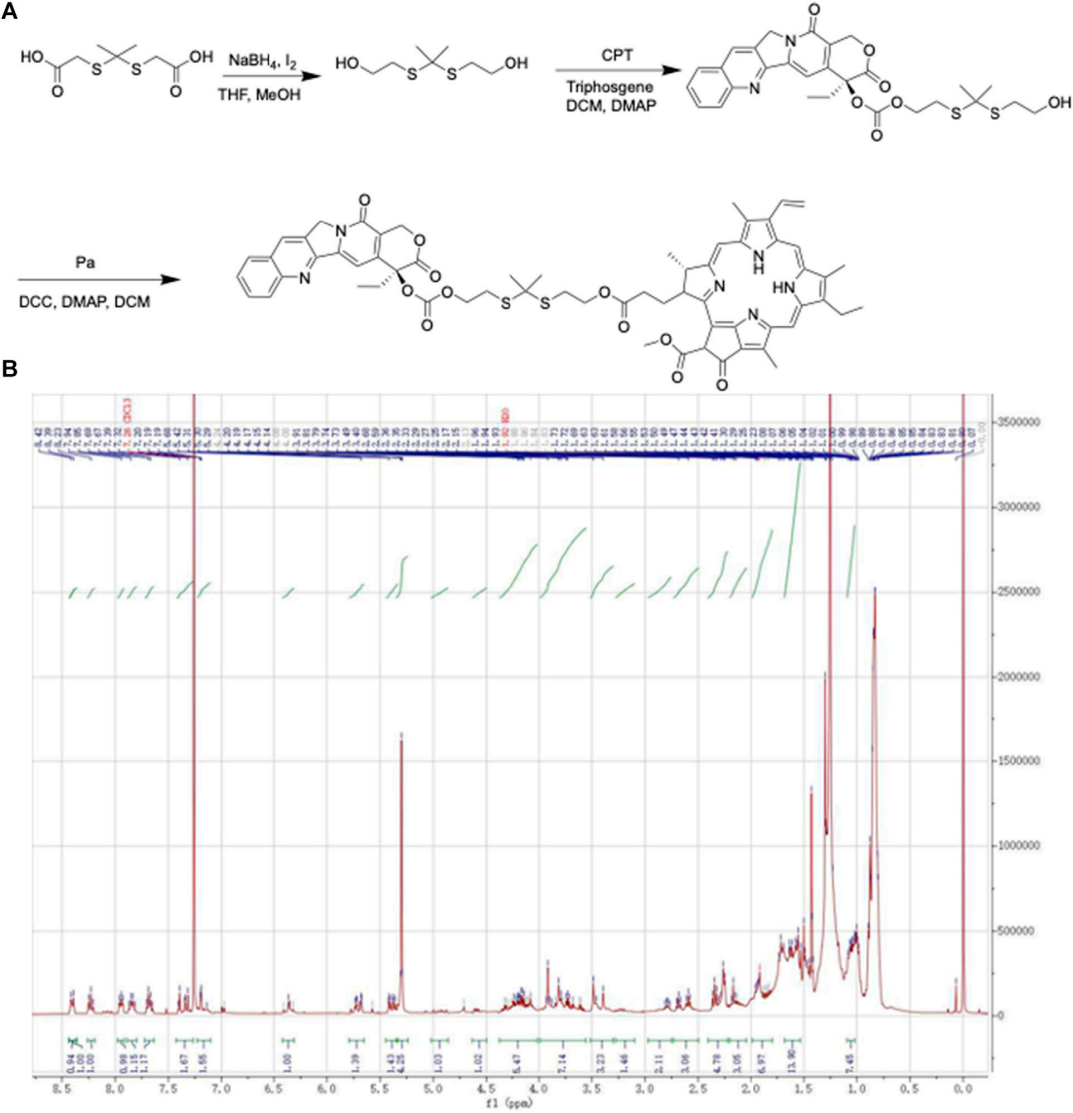
**(A)** Synthesis method and **(B)** ^1^H NMR spectra of CPT-TK-Pa.

The ROS-responsive prodrug, CPT-TK-Pa, was successfully synthesized via 2,2′-[propane-2,2-diylbis (thio)]diacetic acid (Figure 2A). The ^1^H NMR spectra provided evidence for the successful preparation of CPT-TK-Pa, as shown in Figure 2B (400 MHz, chloroform-d): δ 8.40 (d, J = 9.2 Hz, 1H), 8.23 (t, J = 8.6 Hz, 1H), 7.98–7.92 (m, 1H), 7.84 (q, J = 7.7 Hz, 1H), 7.68 (q, J = 7.2 and 6.7 Hz, 1H), 7.42–7.27 (m, 2H), 7.23–7.10 (m, 3H), 6.36 (d, J = 5.7 Hz, 1H), 5.79–5.65 (m, 1H), 5.45–5.33 (m, 1H), 5.30 (s, 4H), 4.18 (ddd, J = 15.4, 11.5, and 7.2 Hz, 5H), 3.99–3.55 (m, 7H), 3.44 (d, J = 36.0 Hz, 3H), 2.79 (dd, J = 11.1 and 6.8 Hz, 2H), 2.64 (dt, J = 35.8 and 7.2 Hz, 3H), 2.41–2.22 (m, 5H), 2.16 (d, J = 8.5 Hz, 3H), 1.99–1.80 (m, 4H), 1.73–1.42 (m, 6H), 1 and .09–0.94 (m, 6H).

### 2.2 Preparation and characterization of the platinum nanozyme

**FIGURE 3:**
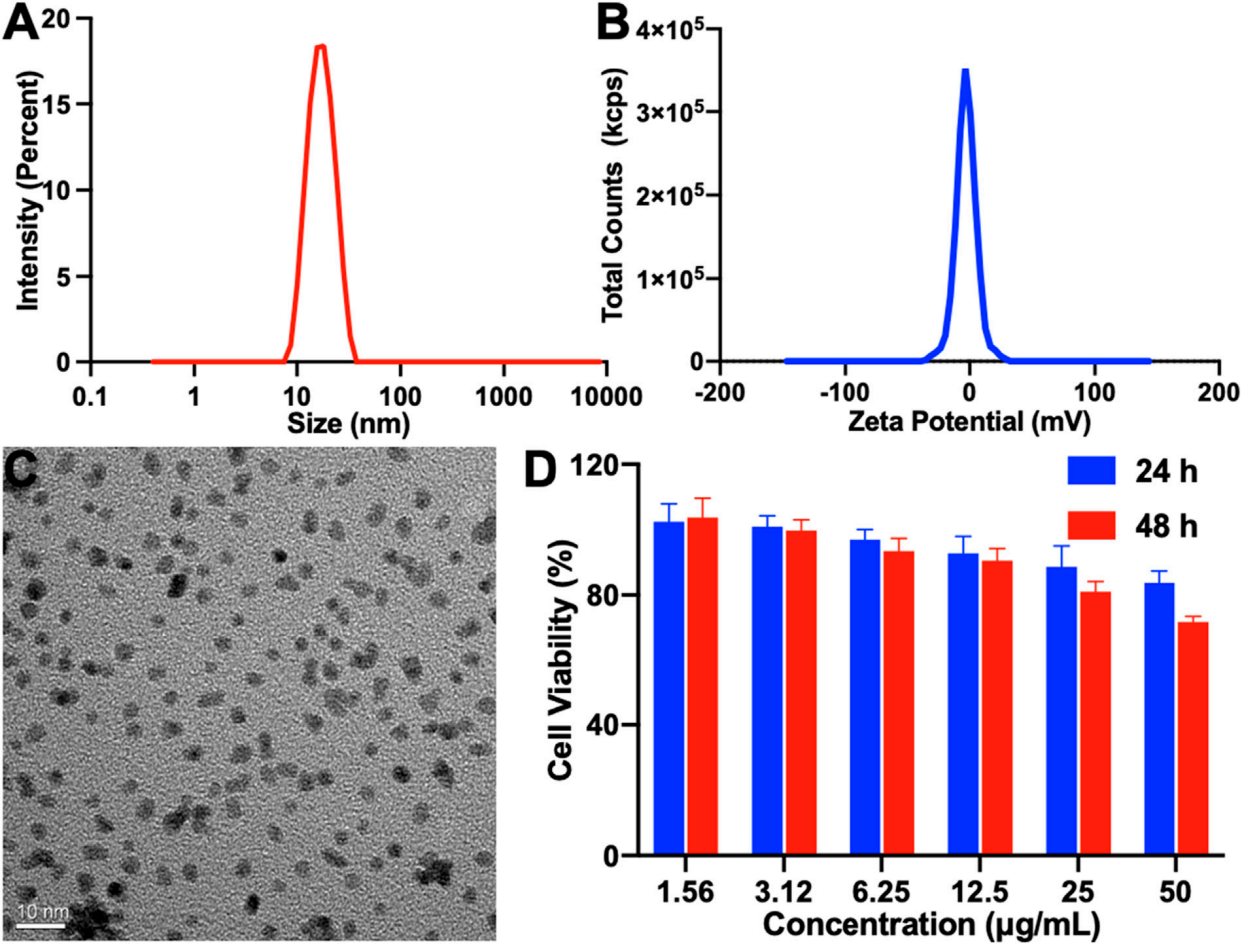
Characterization of PtNP. **(A)** Particle size, **(B)** zeta potential, **(C)** TEM image (scale bar: 10 nm), and **(D)** *in vitro* cell biocompatibility of PtNP.

To enhance the efficacy of photodynamic therapy, we synthesized a platinum nanozyme (PtNP) via a sodium borohydride reduction method (Xu et al., 2018). As illustrated in Figure 3A, the results of dynamic light scattering (DLS) indicated that the PtNP’s hydrodynamic diameter is approximately 15 nm, accompanied by a narrow polydispersity index (PDI). The zeta potential is −3.38 mV (Figure 3B). Transmission electron microscopy (TEM) further confirmed a homogeneous morphology for PtNPs, with a discernible size of approximately 4 nm, as shown in Figure 3C, consistent with the DLS results. Additionally, we assessed the *in vitro* biocompatibility of PtNPs via co-culture with a mouse fibroblast 3T3 cell line, as shown in Figure 3D. Notably, even at a concentration of 50 μg mL^−1^ and after 48 h of incubation, cell viability exceeded 71%, demonstrating that the concentration of the PtNP we used in our study is safe.

### 2.3 Preparation and characterization of polymeric nanoparticles

**FIGURE 4:**
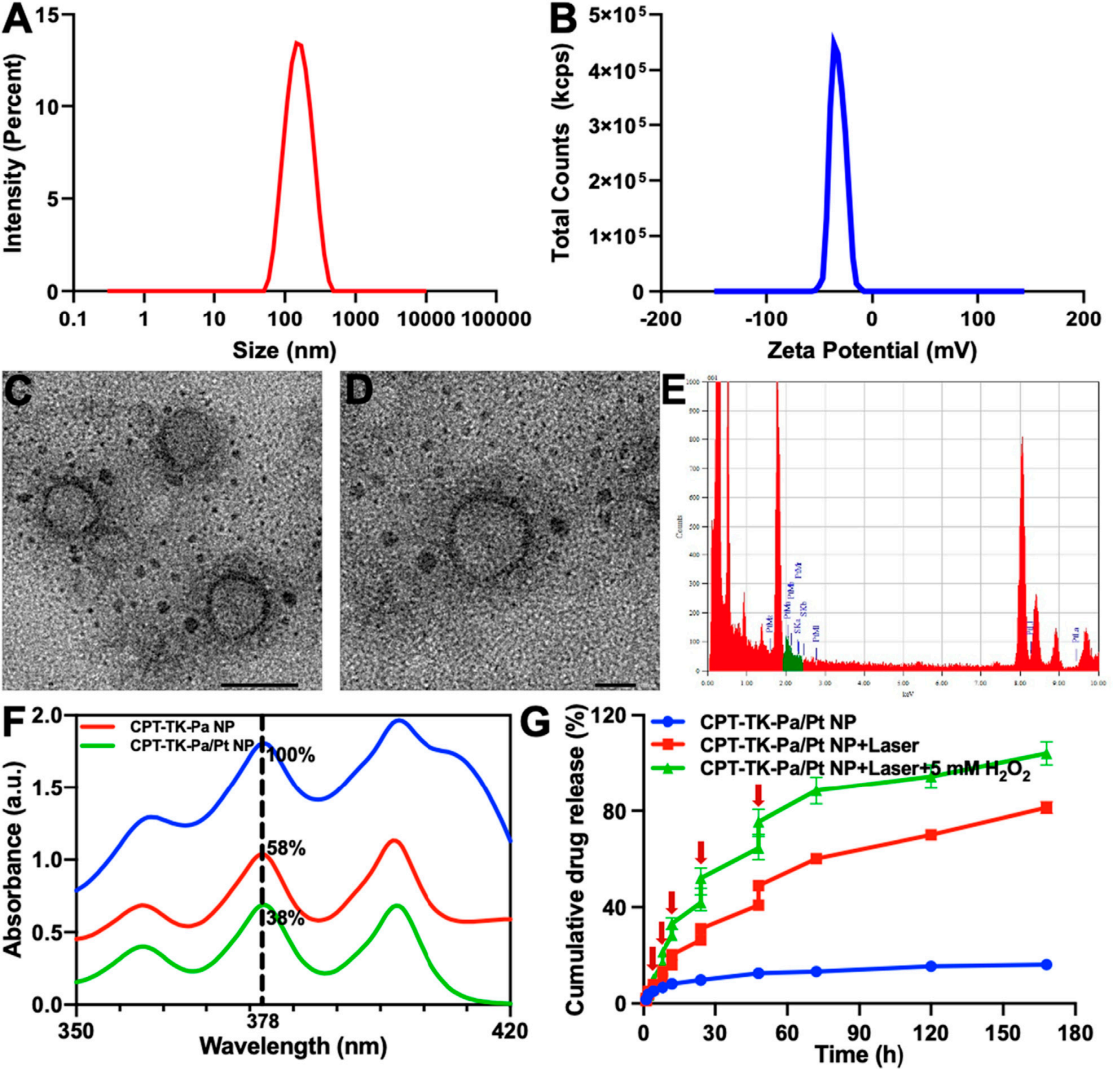
**(A)** Particle size, **(B)** zeta potential, **(C)** TEM image (scale bar: 50 nm), **(D)** localized enlarged TEM image (scale bar: 20 nm), and **(E)** localized region energy dispersive X-ray spectroscopy of CPT-TK-Pa/Pt NP. **(F)** UV-vis absorption spectra of ABDA in the control (blue), CPT-TK-Pa NP (red), and CPT-TK-Pa/Pt NP (green) groups with 100 µM H_2_O_2_ at the designated time with 660 nm laser irradiation. **(G)** Release situations of CPT-TK-Pa/Pt NP under different conditions (the red arrow represents laser irradiation).

The prodrug CPT-TK-Pa and PtNPs were encapsulated in the amphiphilic polymer DSPE–PEG to form the polymeric nanoparticle CPT-TK-Pa/Pt NP. The hydrodynamic diameter of the nanoparticle was 142 nm (Figure 4A), and its zeta potential was recorded at −34 mV (Figure 4B). DSPE–PEG is known for its capacity to prolong both circulation and tumor retention time (Che et al., 2015; Gao et al., 2023), thus allowing nanoparticulate drugs sufficient time and opportunity to cross the endothelial barrier of tumor vessels via the enhanced permeability and retention (EPR) effect (Wang et al., 2023) and directly access the tumor matrix. TEM confirmed a uniform nanoparticle size of approximately 60 nm (Figures 4C,D). Furthermore, energy dispersive X-ray spectroscopy of a localized region with the CPT-TK-Pa/Pt NP (Figure 4E) substantiated that the PtNP was coated on the outer layer of the nanoparticle, in accordance with the intended design.

### 2.4 ROS production *in vitro*

The ROS level is an important determinant of the efficacy of photodynamic therapy. 9,10-Anthracenediyl-bis(methylene)-dimalonic acid (ABDA) has been used as a measure of *in vitro* ROS production (Sztandera et al., 2022). Since ROS-mediated oxidation decreases the absorption peak of ABDA at 378 nm, this property was used to analyze ROS from CPT-TK-Pa NP and CPT-TK-Pa/Pt NP under 660 nm laser irradiation (200 mW cm^−2^). We quantitatively captured the UV-vis absorbance changes at 378 nm, as shown in Figure 4F, after 5 min exposure to a 660 nm laser, the absorbances of CPT-TK-Pa NP and CPT-TK-Pa/Pt NP decreased to 58% and 38%, respectively, highlighting that the presence of PtNP could enhance the ROS production ability of CPT-TK-Pa/Pt NP. This enhancement was attributed to the additional oxygen derived from H_2_O_2_ decomposition. Thus, in accordance with our previous research (Hao et al., 2020) and initial hypotheses, PtNP demonstrates the ideal capacity to enhance the ROS generation ability of CPT-TK-Pa/Pt NP.

### 2.5 Light-responsive drug release characteristics

The thioether bond embedded in the prodrug exhibits a pronounced oxidative responsiveness. Therefore, the release characteristics of the nanoparticles in the presence and absence of H_2_O_2_ were evaluated using 660 nm irradiation. As presented in Figure 4G, in the absence of external stimuli, CPT-TK-Pa/Pt NP displayed a slow and gradual release pattern with only 18% after 168 h, indicating the inherent stability of the nanoparticle. Under 660 nm irradiation, the drug release behavior could be accelerated, and there was a significant increase in the release behavior of CPT-TK-Pa/Pt NP with or without H_2_O_2_, with approximately 20% and 33% after 12 h, respectively, highlighting a light-responsive release mechanism. Furthermore, incorporation of 5 mM H_2_O_2_ along with 660 nm laser exposure activated the release kinetics, resulting in nearly complete drug release (−100%) after 168 h. This demonstrated the capability of the CPT-TK-Pa/Pt NP for precise, light-responsive therapeutic delivery.

### 2.6 Cell uptake study

**FIGURE 5:**
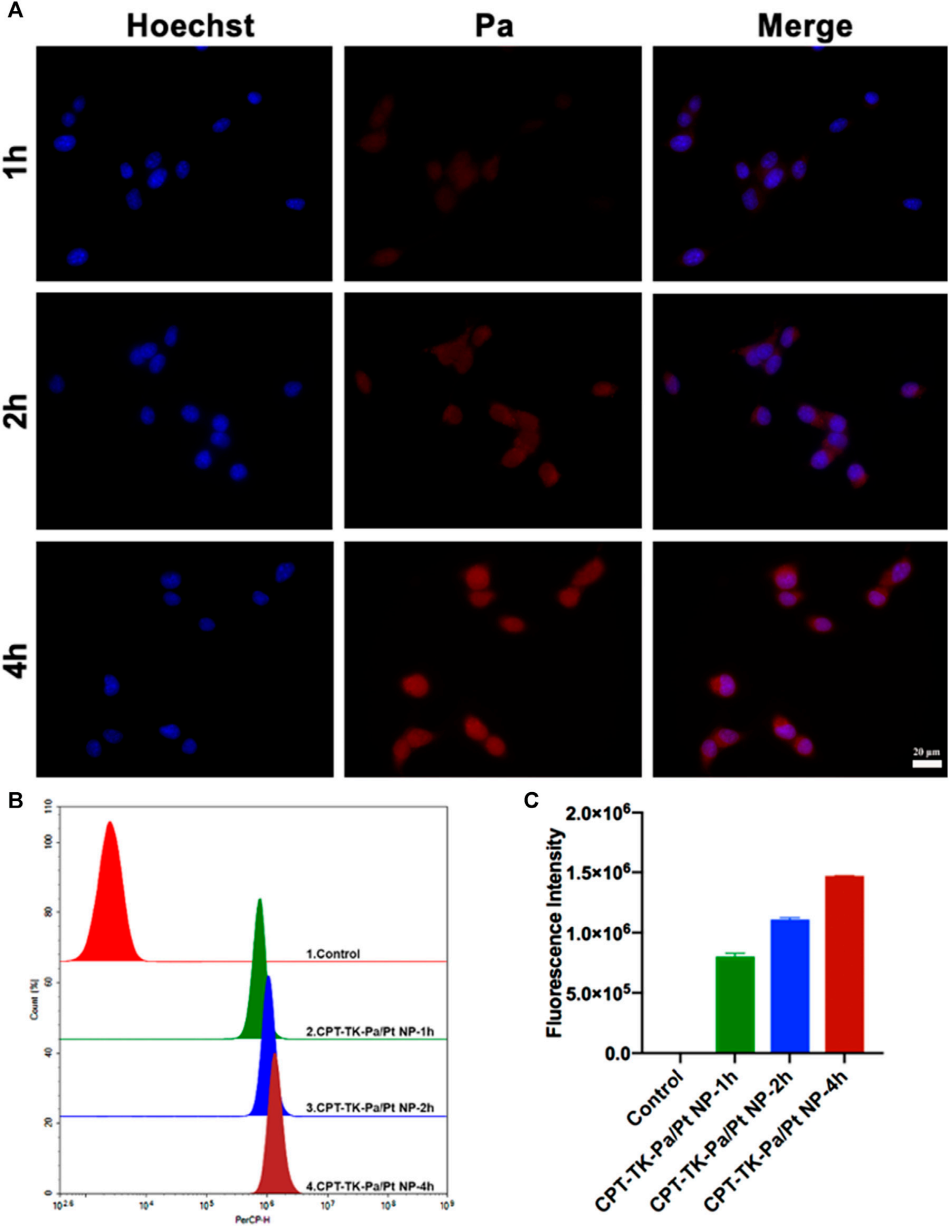
**(A)** Fluorescence micrographs (scale bar: 20 µm), **(B)** flow cytometry analysis, and **(C)** quantitative fluorescence intensity of cell uptake ability of CT26 cells.

Subsequently, we assessed the cell uptake ability of CPT-TK-Pa/Pt NP via co-culture with mouse colon carcinoma CT26 cells (Han et al., 2019). The time course of cell uptake of CPT-TK-Pa/Pt NP was examined within 4 h (Figure 5). After 1 h of incubation, the Pa channel showed a weak red fluorescence signal. However, after 4 h, a distinct red fluorescence signal surrounded the blue Hoechst 33342-stained cell nuclei. These phenomena indicated that the CPT-TK-Pa/Pt NP underwent time-dependent cellular internalization. We further quantified the intracellular fluorescence after CPT-TK-Pa/Pt NP treatment using flow cytometry, and the results were consistent with the above fluorescence microscopy findings.

### 2.7 Intracellular ROS level

**FIGURE 6:**
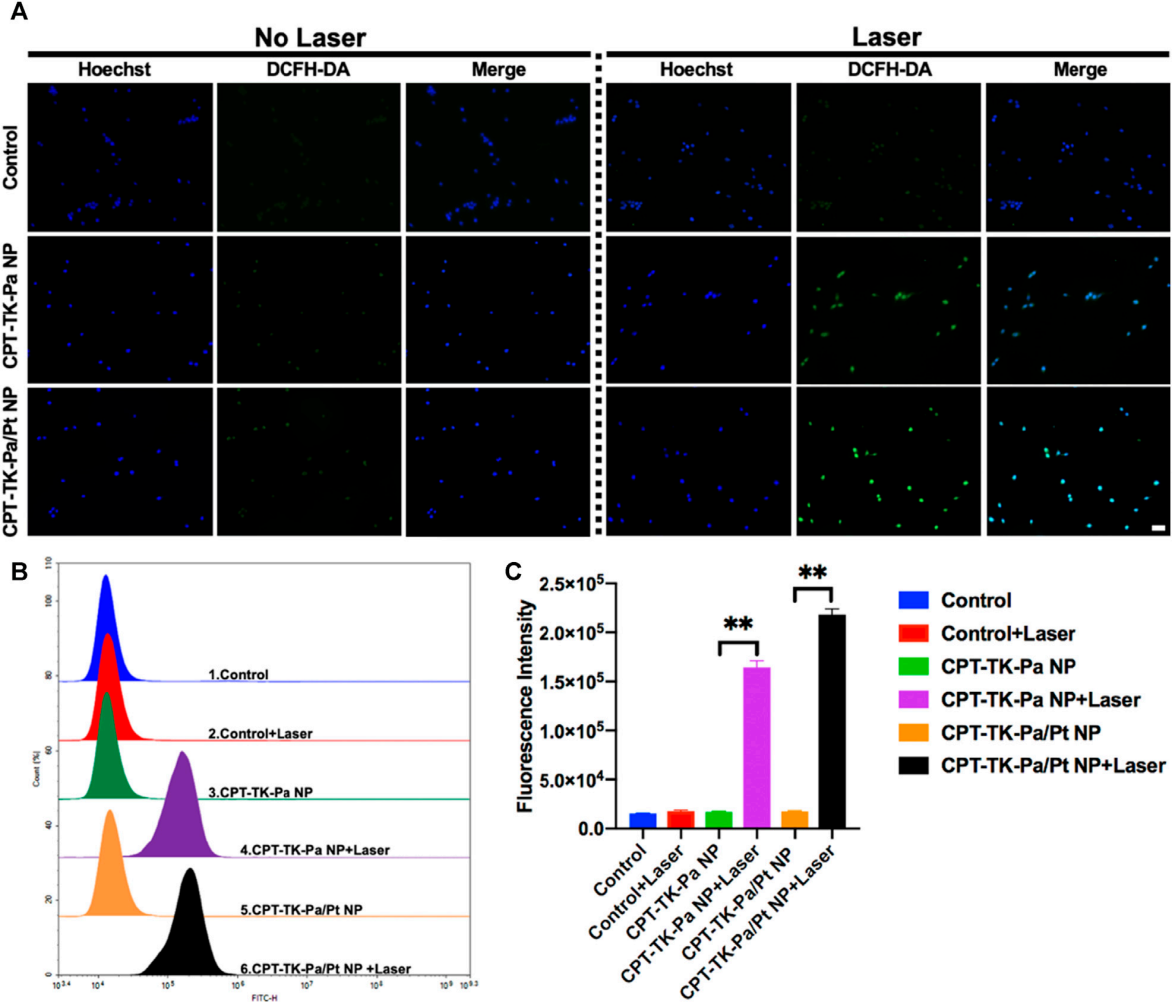
**(A)** Fluorescent micrographs of intracellular ROS levels, employing DCFH-DA as the probe (blue channel: Hoechst; green channel: DCFH-DA; scale bar: 50 µm). **(B)** Flow cytometry analysis of ROS generation and **(C)** quantitative assessment of intracellular ROS synthesis.

Based on the preliminary *in vitro* study of ROS generation, we used a fluorescent probe, 2′,7′-dichlorofluorescin diacetate (DCFH-DA) (Eruslanov and Kusmartsev, 2010), to quantify intracellular ROS levels. Intrinsically, DCFH-DA is non-fluorescent; however, cellular ROS can oxidize DCFH to DCF, rendering it fluorescent. The untreated control group exhibited minimal fluorescence with no significant change upon 660 nm irradiation (Figure 6A). Groups treated with CPT-TK-Pa NP and CPT-TK-Pa/Pt NP, without exposure to 660 nm wavelength illumination, also showed weak fluorescence signals. Upon illumination of CPT-TK-Pa NP and CPT-TK-Pa/Pt NP (parameters: 660 nm and 200 mW cm^−2^) for 5 min, intense intracellular fluorescence was detected in the DCFH-DA channel, indicating that both CPT-TK-Pa NP and CPT-TK-Pa/Pt NP promoted intracellular ROS synthesis, which is helpful in photodynamic therapy. It is noteworthy that the ROS concentration of CPT-TK-Pa/Pt NP surpassed that of CPT-TK-Pa NP, a phenomenon attributed to the PtNP facilitating enhanced oxygen availability for ROS synthesis. Furthermore, we conducted a quantitative ROS assessment by flow cytometry, as presented in Figures 6B,C. The results confirmed that under illumination at a wavelength of 660 nm, the ROS generation levels for CPT-TK-Pa NP and CPT-TK-Pa/Pt NP were increased by 9.6-fold and 12.3-fold, respectively, compared to their unirradiated controls. The results were in congruence with the imaging data presented, which, consistent with our previous findings, further demonstrated that Pa could generate ROS for PDT and that the introduction of PtNP could generate more oxygen to enhance the PDT effect (Hao et al., 2020).

### 2.8 *In vitro* cytotoxicity study

**FIGURE 7:**
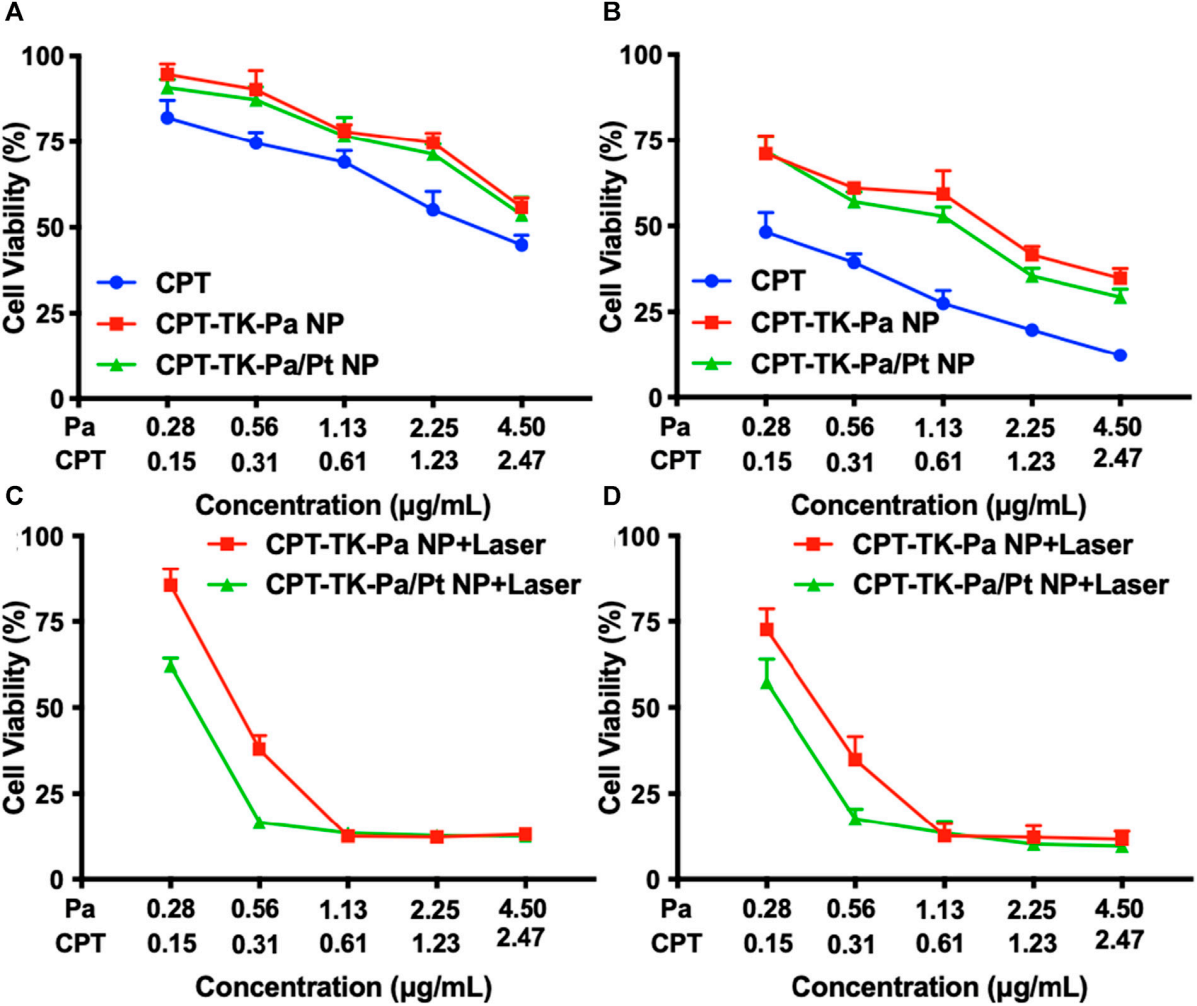
Cell viability of CT26 cells after different treatments. Treatment with CPT, CPT-TK-Pa NP, and CPT-TK-Pa/Pt NP for **(A)** 24 and **(B)** 48 h. Treatment with CPT-TK-Pa NP + laser and CPT-TK-Pa/Pt NP + laser for **(C)** 24 and **(D)** 48 h incubation.

The toxic effects of various nanoparticles, with or without exposure to a 660 nm light, were evaluated on CT26 cell lines using the 3-(4,5-dimethyl-2-thiazolyl)-2,5-diphenyl-2-H-tetrazolium bromide (MTT) assay (Ghasemi et al., 2023). Toxicity assays were first performed on CPT, CPT-TK-Pa NP, and CPT-TK-Pa/Pt NP after 24 and 48 h of incubation with CT26 cells (Figures 7A,B). Incubated for either 24 or 48 h, in the absence of 660 nm laser irradiation, CPT exhibited stronger cytotoxic effects than CPT-TK-Pa NP and CPT-TK-Pa/Pt NP groups in a dose-dependent manner, which was due to the delayed intracellular release of CPT from the prodrugs in the absence of 660 nm laser irradiation. Additionally, cytotoxicity under a 660 nm laser was also investigated. CPT-TK-Pa NP and CPT-TK-Pa/Pt NP exhibited pronounced cytotoxicity under illumination (660 nm and 200 mW cm^−2^) for 5 min (Figures 7C,D). After 48 h of incubation, the cell viability rates for the abovementioned groups were 34.88% and 17.66%, which correlated with the corresponding concentrations of CPT and Pa at 0.31 and 0.56 μg mL^−1^, respectively, indicating that enough ROS generation by CPT-TK-Pa NP and CPT-TK-Pa/Pt NP could sufficiently eradicate cells and accelerate CPT release, rendering it useful for chemophotodynamic therapy of colon carcinoma. Significantly, the inclusion of PtNP in the CPT-TK-Pa/Pt NP groups enhanced the cytotoxic results compared to CPT-TK-Pa NP with 660 nm laser exposure, proving the ability of PtNP to enhance the oxygen level and strengthen the photodynamic efficacy of polymeric nanoparticles.

### 2.9 Cell apoptosis analysis

**FIGURE 8:**
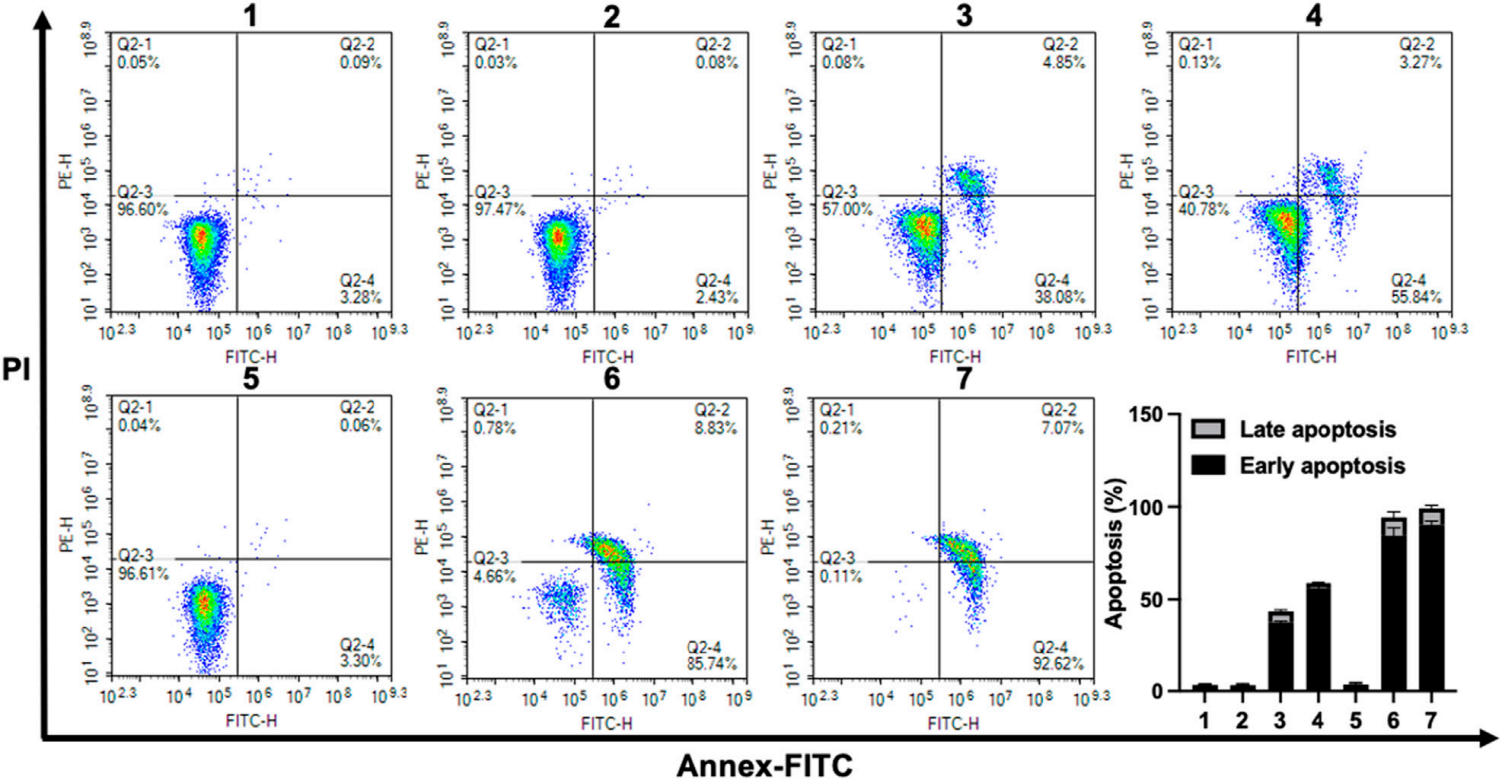
Flow cytometric assessment of CT26 cell apoptosis after different treatments. (1. control, 2. Pa, 3. CPT-TK-Pa NP, 4. CPT-TK-Pa/Pt NP, 5. control + laser, 6. CPT-TK-Pa NP + laser, and 7. CPT-TK-Pa/Pt NP + laser).

Cellular apoptosis induced by Pa, CPT-TK-Pa NP, and CPT-TK-Pa/Pt NP, all in the presence and absence of 660 nm laser irradiation, was investigated utilizing the Annexin V-FITC apoptosis detection protocol (Wallberg et al., 2016). The apoptosis rates of the CPT-TK-Pa NP and CPT-TK-Pa/Pt NP groups with 660 nm irradiation exceeded those observed in their non-exposed counterparts, suggesting the ability of the laser to amplify the antineoplastic efficacy of the nanoparticles (Figure 8). Importantly, among the groups, the CPT-TK-Pa/Pt NP subset with 660 nm laser exposure registered the highest levels of apoptosis, up to 90.35%. This was in line with the *in vitro* cytotoxicity study and suggested that the light-responsive release of CPT, coupled with the strengthened ROS-generating ability of PtNP within CPT-TK-Pa/Pt NP, enhanced the chemophotodynamic therapeutic approach against colon carcinoma.

## 3 Conclusion

In summary, we have engineered a ROS-responsive prodrug nanoparticle (CPT-TK-Pa/Pt NP) incorporating platinum nanozyme to strengthen and collaborate on the effects of PDT and chemotherapy against colon tumors. The PtNP is capable of catalyzing the conversion of H_2_O_2_ to oxygen, thereby amplifying the ROS-generating capacity of Pa. The release of CPT could be modulated by illumination at a wavelength of 660 nm, enabling smart release. Upon 660 nm laser irradiation, the CPT-TK-Pa/Pt NP demonstrated potentiated cytotoxic effects and suppressed tumor cell proliferation. This investigation offers an innovative prospect for clinical therapeutic progress in the colon cancer field.

## 4 Materials and methods

### 4.1 Materials

Sodium borohydride (NaBH_4_), chloroplatinic acid hexahydrate (H_2_PtCl_6_·6H_2_O), and polyvinyl alcohol (PVA) were obtained from Sigma-Aldrich (Saint Louis, Missouri, United States). Pheophorbide a was purchased from J&K Scientific Ltd. (Beijing, China). DSPE-PEG_2000_ was purchased from Shanghai Aladdin Biochemical Technology Co., Ltd. (Shanghai, China). 2,2′-[Propane-2,2-diylbis (thio)]diacetic acid was purchased from Shanghai Bi De Pharmaceutical Technology Co., Ltd. (Shanghai, China). Camptothecin (CPT) was purchased from Meilun Biology Technology Co., Ltd. (Dalian, China). Tetramethylbenzidine (TMB) and hydrogen peroxide (H_2_O_2_) were obtained from Shanghai Titanchem Co., Ltd. (Shanghai, China). The Reactive Oxygen Species Assay Kit and Hoechst 33342 were purchased from Beyotime Biotechnology Co., Ltd. (Shanghai, China). The Annexin V-FITC Apoptosis Detection Kit (BD PharMingen) was obtained from Shanghai Universal Biotechnology Co., Ltd. (Shanghai, China). All the materials used in this article were of analytic grade and used as received.

### 4.2 Synthesis and characterization of CPT-TK-Pa

As shown in Figure 2A, we used NaBH_4_ to reduce the corresponding carboxyl residues of 2,2′-[propane-2,2-diylbis (thio)] diacetic acid to hydroxyl groups. Subsequently, we conducted a reaction with stoichiometric phosgene and the catalyst DMAP to conjugate the drug CPT to a hydroxyl group. Finally, the PA was attached to another hydroxyl group by the esterification reaction to obtain CPT-TK-Pa, and ^1^H NMR spectra (Varian 400 spectrometer, Varian, United States) were used to characterize the prodrug.

### 4.3 Preparation and characterization of the platinum nanozyme

The synthesis of PtNP was performed using a reduction method using sodium borohydride, as reported by Xu et al. (2018). In brief, 1 mL of H_2_PtCl_6_·6H_2_O (10 mM) and PVP (16 mg) were dissolved in 9 mL of water, and a cold solution of NaBH_4_ (4.4 mg) was added with stirring for 4 h at room temperature. Finally, a 100 kDa MWCO electrocatalyst was used to purify the PtNP. DLS and TEM were used to characterize the particle size and morphology of the PtNP. In addition, mouse fibroblast cell line 3T3 cells were used to evaluate the cell viability of PtNP using the MTT method.

### 4.4 Preparation and characterization of polymeric nanoparticles

Initially, the 100 µg prodrug CPT-TK-Pa was solubilized in 1 mL of dichloromethane. Subsequently, 8 µL of PtNP was introduced under sonication using a probe-type ultrasonic processor for 6 min, forming a primary emulsion. This primary emulsion was then combined with 1 mg of DSPE–PEG in a 10 mL water solution and subjected to an additional 6 min of sonication. After removing the organic solvent and any unloaded agents via rotary evaporation under vacuum, the prodrug CPT-TK-Pa and PtNP co-loaded nanoparticle, denoted as CPT-TK-Pa/Pt NP, was successfully synthesized. Comprehensive characterization of the polymeric nanoparticle, including assessments of particle size, zeta potential, and morphology, was conducted using DLS and TEM.

### 4.5 ROS production *in vitro*

Upon illumination with a 660 nm laser at an intensity of 200 mW cm^−2^, the ROS production capability of the nanoparticles was assessed by ABDA’s absorbance shifts at 378 nm (Sztandera et al., 2022). Precisely, a 100 µL aliquot from the ABDA solution (1 mg mL^−1^) was integrated with CPT-TK-Pa NP and CPT-TK-Pa/Pt NP solutions, each standardized to an equivalent of 10 μg mL^−1^ of Pa. These solutions, post-deoxygenation, incorporated a concentration of 1 × 10^−4^ m H_2_O_2_ and were irradiated by the laser with the aforementioned laser parameters. Subsequently, the ROS-producing capability of the nanoparticles was analyzed at predetermined intervals via UV-vis spectroscopy.

### 4.6 Light-responsive drug release characteristics

The light-responsive drug release characteristics of the nanoparticles were explored using a modified dialysis approach under 660 nm laser illumination with or without H_2_O_2_. In detail, solutions of CPT-TK-Pa/Pt NP, amounting to 1 mL, were confined within dialysis bags (with a molecular cutoff of 3500). These were immersed in 15 mL of phosphate buffered saline (PBS) with and without 5 × 10^−3^ M H_2_O_2_, followed by gentle agitation at 100 rpm and maintenance at 37°C. For laser-exposed groups, periodic irradiation was executed (660 nm, 200 mW cm^−2^), after which medium exchanges were carried out with fresh and pre-warmed PBS. The CPT concentrations in the collected medium were quantified via high-performance liquid chromatography (HPLC).

### 4.7 Cell uptake study

The cell uptake efficacy of CPT-TK-Pa/Pt NP within CT26 cells was examined. CT26 cells, once seeded at a density of 2 × 10^5^ per well, were treated with CPT-TK-Pa/Pt NP solutions following a 24 h incubation for additional periods of 1, 2, and 4 h. The cells were subjected to a series of PBS washes three times, followed by nuclear staining using Hoechst 33342 (10 μg mL^−1^). Subsequently, the cells were visualized under a fluorescence microscope. Quantitative cellular uptake was assessed using flow cytometry.

### 4.8 Intracellular ROS level

DCFH-DA (Eruslanov and Kusmartsev, 2010) served as a fluorescent probe for the intracellular ROS concentrations in cells treated with CPT-TK-Pa NP and CPT-TK-Pa/Pt NP. Initially, CT26 cells at a density of 1 × 10^5^ were cultured in a 12-well plate for 24 h. Subsequently, 1 mL of either CPT-TK-Pa NP or CPT-TK-Pa/Pt NP was introduced, with the untreated medium serving as a control. Following a 4 h incubation, DCFH-DA (at 1 × 10^−5^ M concentration) was dispensed into each well, allowing an additional 20 min incubation period. In the laser exposed group, the existing medium was exchanged and illuminated (660 nm and 200 mW cm^−2^) for 5 min. Post-treatment, it was subsequently stained with Hoechst 33342 (10 μg mL^−1^) and visualized under a fluorescence microscope. In addition, quantitative intracellular ROS metrics were acquired via flow cytometry.

### 4.9 *In vitro* cytotoxicity study

MTT assays were employed to explore the cytotoxicity of the various treatments (Ghasemi et al., 2023). After a 24 h seeding of CT26 cells at a density of 4 × 10^3^, they were treated with various concentrations of CPT, CPT-TK-Pa NP, and CPT-TK-Pa/Pt NP for another 12 h of incubation, subsequently under a 660 nm laser (200 mW cm^−2^) for 5 min. The standard MTT assay was then conducted at another 12 and 36 h of incubation.

### 4.10 Cell apoptosis analysis

CT26 cells were seeded into a 6-well plate at a density of 2 × 10^5^. Following 24 h incubation, the cells were treated with Pa, CPT-TK-Pa NP, and CPT-TK-Pa/Pt NP solutions on the plates separately. Following a 12 h culture period, these cells were exposed to a 660 nm laser (200 mW cm^−2^) for 5 min or not. Following incubation for an additional 12 h, the cells from each plate were harvested and subsequently stained using the Annexin V-FITC apoptosis detection protocol according to the manufacturer’s standard (Wallberg et al., 2016).

## 5 Statistical analysis

All the resultant data are denoted as the mean ± standard deviation (SD). The statistical analysis was conducted using Prism 8.0 software. The statistical significance was determined using the Student's t-test or one-way ANOVA. Significance thresholds were demarcated at *p* < 0.05 (*) and *p* < 0.01 (**).

## Data Availability

The original contributions presented in the study are included in the article, further inquiries can be directed to the corresponding author.
